# The first Chinese with Hb Chile leading to chronic anemia and methemoglobinemia: a case report

**DOI:** 10.1186/s12887-023-04462-8

**Published:** 2023-12-18

**Authors:** Yao Gong, Qinxin Zheng, Sili Long, Hongying Chen, Wenjun Liu, Cheng Li

**Affiliations:** 1 Department of Pediatrics, the Affiliated Hospital of Southwest Medical University, Luzhou, China; 2 Sichuan Clinical Research Center for Birth Defects, Luzhou, Sichuan 646000 China

**Keywords:** Hemoglobin variant, Unstable hemoglobin, Hemoglobin Chile, Methemoglobinemia, anemia

## Abstract

**Background:**

Hemoglobin (Hb) Chile [β28(B10) Leu > Met; HBB: c.85 C > A] is a rare hemoglobin variant caused by a missense mutation in the HBB gene. Only one case of Hb Chile has been reported worldwide so far. It is an unstable hemoglobin, characterized by cyanosis associated with chronic methemoglobinemia and hemolytic anemia induced by sulfonamides or methylene blue.

**Case presentation:**

A 9-year-3-month-old girl had mild anemia of unknown etiology for more than 6 years. She had a slight pallor without other symptoms or signs. The complete blood count revealed normocytic normochromic anemia with a sometimes-elevated reticulocyte count, and the bone marrow cytology showed marked erythroid hyperplasia, but the tests related to hemolysis were normal. Therefore, the whole exome sequencing was performed and showed a heterozygous mutation for HBB: c.85 C > A. With asymptomatic methemoglobinemia confirmed later, she was eventually diagnosed with Hb Chile.

**Conclusions:**

This is the first report of Hb Chile in China and the second worldwide. This case shows that Hb Chile is clinically heterogeneous and difficult to diagnose and expands our understanding on the clinical and hematological traits of the disease.

**Supplementary Information:**

The online version contains supplementary material available at 10.1186/s12887-023-04462-8.

## Background

Hemoglobin variants are variants of HbA, HbA2, or HbF caused by a mutation in the globin gene, resulting in a change in the primary structure of the globin chain [1]. Some variants can significantly alter the physicochemical properties, stability, oxygen affinity, synthesis of the hemoglobin molecule, or cause autoxidation of heme ferrous iron. Consequently, individuals with such variants may exhibit hematological abnormalities and/or clinical manifestations.

Hb Chile is a rare variant, first mentioned in a personal communication from Dr. H.P. Seelig to Professor Dr. T.H.J. Huisman, but no details have been released. Later, the first case report of Hb Chile was published by R. Hojas-Bernal et al. in 1999, which presented with chronic cyanosis associated with methemoglobinemia and acute hemolytic anemia induced by sulfonamides or methylene blue. It is an unstable hemoglobin, caused by a missense mutation in the HBB gene (HBB: c.85 C > A), resulting in an amino acid substitution of the β-globin chain (β28(B10) Leu > Met) [2]. In this paper, we report the first case of Hb Chile in China and the second worldwide, which is characterized by chronic hemolytic anemia unrelated to drugs and methemoglobinemia without cyanosis.

## Case presentation

A 9-year-3-month-old girl visited the hospital because of “anemia for more than 6 years”. She was incidentally found to have persistent mild anemia at the age of 3, with slight pallor, without bleeding, jaundice, abnormal urine color, bone pain, hepatosplenomegaly, or superficial lymphadenopathy. She did not suffer from chronic kidney disease, chronic liver disease, endocrine disease, or connective tissue disease. Moreover, her family members are free from anemia. Since the age of 6, she had been to several hospitals for anemia. The complete blood count (CBC) revealed normocytic normochromic anemia with a sometimes-elevated reticulocyte count (Table 1), and the bone marrow cytology showed marked erythroid hyperplasia without morbid hematopoiesis. Therefore, she was suspected to have congenital hemolytic anemia. However, the tests related to hemolysis were normal (Table 2). In such situations, we performed trios whole exome sequencing (WES) with parents’ informed consent. The WES showed a heterozygous mutation for HBB: c.85 C > A, derived from her mother, which is an unstable hemoglobinopathy: Hb Chile. Then, the arterial blood gas analysis and pulse oximeter saturation for her and her mother were performed (Table 3). It suggested that their methemoglobin was abnormally high and the patient’s pulse oximeter saturation was abnormally low.

**Table 1:** Complete blood count of case

Age	78 months	96 months	110 months	Reference value
WBC (10^9^ /L)	7.81	7.4	6.44	8–10
RBC (10^12^ /L)	3.44	3.33	3.42	4.0-4.5
HB (g/L)	102	99	103	120–140
HCT (%)	32.1	30.6	32.2	35–45
MCV (fl.)	93.3	91.9	94.0	80–94
MCH (pg)	29.7	29.7	30.2	28–32
MCHC (g/L)	318	324	321	320–380
RET (%)	0.58	2.71	-	0.5–1.5
PLT (10^9^ /L)	352	403	320	100–300

Note: WBC, white blood cells; RBC, red blood cells; HB, Hemoglobin; HCT, hematocrit; MCV, mean corpuscular volume; MCH, mean corpuscular hemoglobin; MCHC, mean corpuscular hemoglobin concentration; RET, reticulocytes; PLT, platelets

**Table 2:** Tests related to hemolysis of case

Variables	Results	Reference value
Coomb’s test	Negative	Negative
Isopropanol precipitation assay	Negative	Negative
G-6-PD	5.00 ugHG	> 3.8 ugHG
Hb electrophoresis		
HbA HbF HbA2 Abnormal Hb	97.2% 0.0% 2.8% 0.0%	≥ 94.5% 0.0-2.0% 2.3-3.2% 0.0%
Thalassemia-gene	No mutation was found (23 common mutations of thalassemia in Chinese)	-
Blood biochemistry	No significant elevations in bilirubin and LDH	-
Abdominal ultrasound	No hepatosplenomegaly or gallstone	-

**Table 3:** Arterial blood gas analysis and pulse oximeter saturation

	Daughter	Mother	Reference value
HGB (g/L)	101	125	120–175
MetHb (%)	12.0	2.1	< 1.5
PaO2 (mm Hg)	87.5	91.5	83–108
SaO2 (%)	89.8	97.2	93–98
Pulse oximeter saturation (%)	74–79	98–99	95–100

## Discussion and conclusions

Unstable hemoglobin is due to mutations in the globin chain that alter hemoglobin stability, leading to red blood cells that are easier captured by the spleen and shortened lifespan [3]. At present, 156 types of unstable hemoglobin have been reported [4]. Clinical manifestation ranges from asymptomatic hemolysis to compensatory hemolysis to severe anemia, some with methemoglobinemia. Hemolysis tests reveal nonspecific hemolytic features. Heinz body stain test, heat denaturation test, and isopropanol precipitation assay could be used as screening tests for unstable hemoglobin. Hemoglobin electrophoresis or high-performance liquid chromatography (HPLC) cannot reliably detect all unstable hemoglobin [5]. Therefore, genetic testing is the most definitive means of determining the diagnosis and characterizing the variants. Human blood containing more than 1% methemoglobin (MetHb) is called methemoglobinemia, and clinical presentations include hypoxia, cyanosis, and erroneous pulse oximeter saturation. Methemoglobinemia caused by the Hb variant is often detected due to cyanosis or abnormal pulse oximetry readings without obvious signs of hypoxia [6–9].

**Fig. 1:**
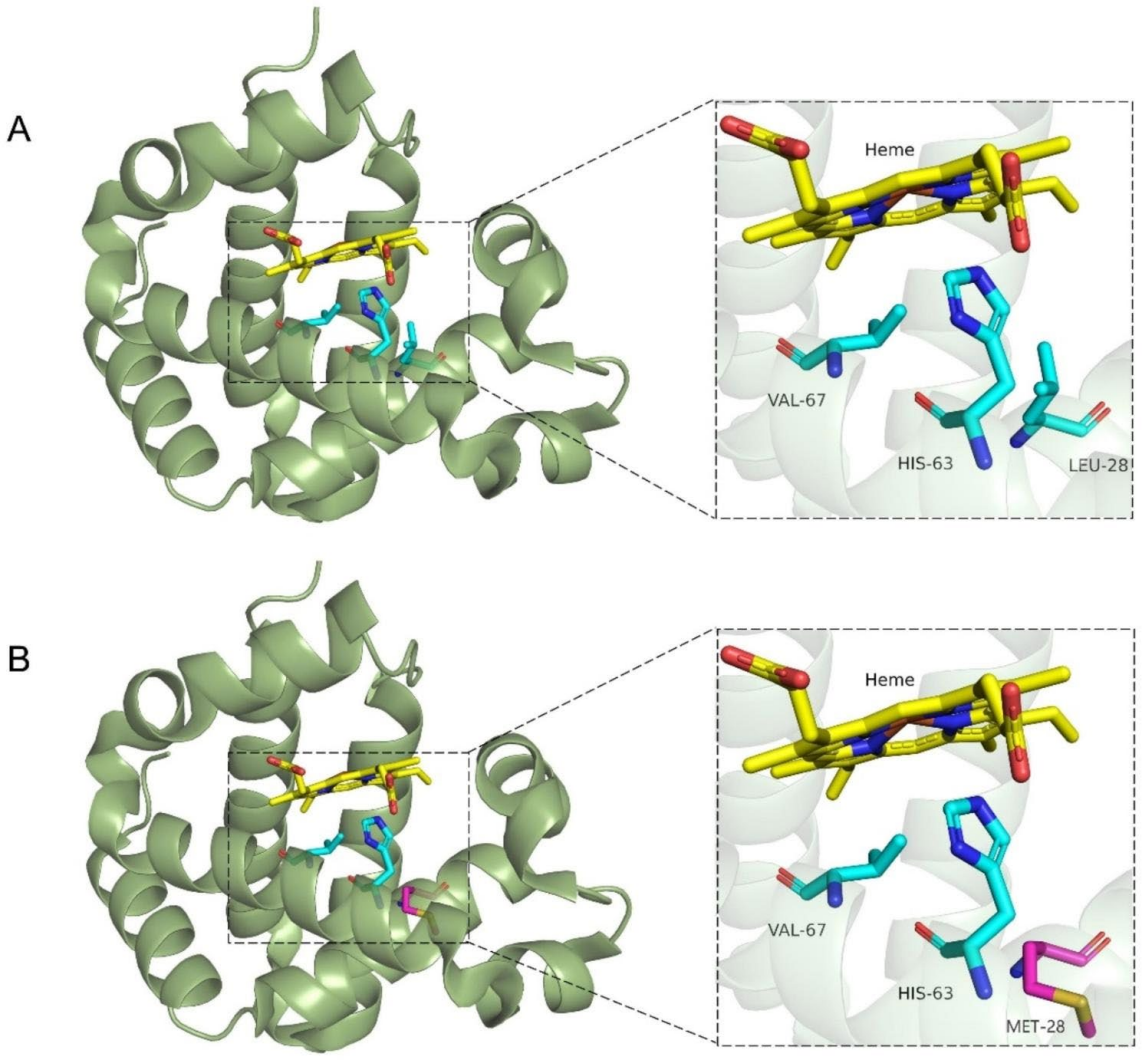
**A**: Diagram of the distal heme pocket of β-chain of wild-type. **B**: Diagram of the distal heme pocket of β-chain of Hb Chile. Diagrams are generated from the PyMOL program, and data is obtained from PDB entry 1ZX2.

So far, there is no data on the crystal structure of Hb Chile and no clear molecular pathological mechanism to explain its instability and methemoglobina. We summarize the clinical phenotypes of variants substituted at amino acid 28 (both at residue 10 of the B helix) of the β-globin and γ-globin peptide chains by the literature curation in Table 4 [2, 10–13]. It shows that the substitution of the amino acid at this position could lead to unstable hemoglobin and/or methemoglobinemia [2]. Because the β28(B10) is located in a significant area of the distal heme pocket as shown in Fig. 1, we speculate that the amino acid substitution here may reduce the stability of heme-globin binding and eventually make the globin unstable. On the other hand, it may also alter the hydrophobicity of the heme pocket leading to oxidation of divalent ferrous (Fe2+) to trivalent ferric (Fe3+) in heme [1, 14]. All three individuals with the mutation (HBB: c. 85 C > A) had methemoglobinemia, but their hemolytic anemia showed heterogeneity. The first case developed acute hemolytic anemia only upon exposure to oxidizing drugs, while the girl in this paper presented with chronic hemolytic mild anemia unrelated to the drug, and her mother had no anemia. We speculate that heterogeneity may be related to the co-inheritance other hemoglobin variants, the ability of erythrocyte reducing, and the functional status of the spleen [15]. For instance, previous studies have demonstrated that unstable Hb Rush [β101 (G3) Glu > Gln; HBB: c.304G > c] co-inherited with Hb E or β-thalassemia increases the proportion of Hb Rush and manifests as thalassemia intermedia. However, individuals who are heterozygous for Hb Rush alone typically exhibit mild hemolytic anemia [16]. It has also been shown that unstable Hb-Haná [β63 (E7) His > Asn; HBB: c.190 C > A] alone generally does not cause hemolytic anemia. However, combined with partial glutathione reductase (GR) deficiency, it will reduce the antioxidant capacity of red blood cells, which leads to Heinz body hemolytic anemia [15]. But, none of the three individuals with Hb Chile co-inheritance other hemoglobin variants. In addition, it is unclear whether the first case had a GR gene mutation, which was not present in the girl and her mother (not seen on WES).

**Table 4:** Information of variants substituted at amino acid 28 of the β-globin and γ-globin

variants	Amino acid mutation	Gene mutation	Clinical phenotypes
Hb F-M Viseu	γ28(B10) Leu > Met	HBG2:c.85 C > A	Methemoglobinemia, Unstable (10)
Hb Chile	β28(B10) Leu > Met	HBB:c.85 C > A	Methemoglobinemia, Unstable (2)
Hb Saint Louis	β28(B10) Leu > Gln	HBB:c.86T > A	Methemoglobinemia, Unstable (11)
Hb Chesterfield	β28(B10) Leu > Arg	HBB:c.86T > G	Extremely unstable (12)
Hb Genova	β28(B10) Leu > Pro	HBB:c.86T > C	Unstable (13)

This case was very difficult to diagnose based on the following: First, the isopropanol precipitation assay was negative, but some patients with unstable hemoglobinopathies have a negative isopropanol precipitation assay [17]. Second, no abnormal Hb was detected in hemoglobin electrophoresis, but Hb Chile cannot be separated from Hb A by alkaline electrophoresis, acidic agar electrophoresis, IEF electrophoresis, and weak cation exchange HPLC [2]. Third, methemoglobinemia and low pulse oximeter saturation were not perceived. Although the girl had a high level of MetHb (12%), her content was 1.212 g/dL < 1.5 g/dL, so there was no significant cyanosis [6]. In addition, the girl had not previously undergone pulse oximetry and arterial blood gas analysis.

In summary, this is the first report of Hb Chile in China. It is generally not necessary to treat mild anemia and methemoglobinemia, but the patient should pay attention to prevent infection and avoid oxidizing drugs that could aggravate hemolysis [3, 6]. Before the definite diagnosis, her parents were very anxious about her anemia, and visited several hospitals for repeated examinations and gave her various nutritional supplements by themselves. After the definite diagnosis, they reassured the concerns and stopped ineffective treatment. So, it should be diagnosed as early as possible to relieve the anxiety of the patient and her family, so that the patient can be properly treated.

### Electronic supplementary material

Below is the link to the electronic supplementary material.

Supplementary Material 1: CARE Checklist of information to include when writing a case report

Supplementary Material 2: Sequencing data of the patient

## Data Availability

The datasets used and/or analyzed during the current study are available from the corresponding author on reasonable request.

## Abbreviations

Hb: Hemoglobin
CBC: Complete blood count
WES: Whole exome sequencing
HPLC: High-performance liquid chromatography
MetHb: Methemoglobin
GR: Glutathione reductase
