# Supplementary material for: The first Chinese with Hb Chile leading to chronic anemia and methemoglobinemia: a case report
Source: BMC Pediatr. 2023 Dec 18;23:639. doi: 10.1186/s12887-023-04462-8 (PMC10726640; doi:10.1186/s12887-023-04462-8)
Supplement: Supplementary file 2 — Supplementary Material 2: Sequencing data of the patient [file 12887_2023_4462_MOESM2_ESM.doc]

Supplement Table 1 Sequencing data of the patient

| Item | Description |
| --- | --- |
| Project name | Trios‐based whole exome sequencing *V*4 |
| Capture probe | GenCap ® whole exome gene capture probe*V*4.0 (MyGenostics, Beijing, China) |
| Targeted region | Each exon (excluding non-coding regions such as promoters), as well as point mutations and small deletion insertion mutations in the intron of 20 bp adjacent to the exon |
| Average sequencing depth | 123.72× |
| coverage of target regions (20×) | 96.46% |
| Number of total reads | 217 |
| Number of reads carrying the variant | 93 |
